# Health-related quality of life in children and adolescents with Marfan syndrome or related disorders: a controlled cross-sectional study

**DOI:** 10.1186/s13023-024-03191-0

**Published:** 2024-04-30

**Authors:** Thomas Edouard, Marie-Christine Picot, Fernanda Bajanca, Helena Huguet, Aitor Guitarte, Maud Langeois, Bertrand Chesneau, Philippe Khau Van Kien, Eric Garrigue, Yves Dulac, Pascal Amedro

**Affiliations:** 1 Reference Center for Marfan Syndrome and Related Diseases, Children’s Hospital, Toulouse University Hospital, RESTORE, INSERM U1301, Paul Sabatier University, Toulouse, France; 2 Epidemiology and Clinical Research Department, Montpellier University Hospital, Montpellier, France; 3 Medical Genetics Unit, Nîmes University Hospital, Nîmes, France; 4 Department of Pediatric and Adult Congenital Cardiology, M3C National Reference Centre, Bordeaux University Hospital, IHU Liryc, INSERM 1045, University of Bordeaux, Bordeaux, France; 5 Endocrine, Bone Diseases, and Genetics Unit, Children’s Hospital, Toulouse University Hospital, 330 Avenue de Grande-Bretagne TSA 70034, Toulouse Cedex 9, 31059 France

**Keywords:** Marfan syndrome, Health-related quality of life, Aerobic physical fitness, Peak oxygen consumption, Physical well-being, Psychological well-being

## Abstract

**Background:**

This cross-sectional controlled study aims to assess health-related quality of life (HRQoL) of children and adolescents with a molecular diagnosis of Marfan syndrome (MFS) or related disorders and to evaluate the factors associated with HRQoL in this population. Sixty-three children with MFS and 124 age- and sex-matched healthy children were recruited. HRQoL was assessed using the Pediatric Quality of Life Inventory (PedsQL™) generic questionnaire. The correlation between HRQoL scores and the different continuous parameters (age, body mass index, disease severity, systemic score, aortic sinus diameter, and aerobic physical capacity) was evaluated using Pearson’s or Spearman’s coefficient. A multiple linear regression analysis was performed on the two health summary self-reported PedsQL™ scores (physical and psychosocial) to identify the factors associated with HRQoL in the MFS group.

**Results:**

Except for emotional functioning, all other domains of HRQoL (psychosocial and physical health, social and school functions) were significantly lower in children with MFS compared to matched healthy children. In the MFS group, the physical health summary score was significantly lower in female than in male patients (self-report: absolute difference [95%CI] = -8.7 [-17.0; -0.47], *P* = 0.04; proxy-report: absolute difference [95%CI] = -8.6 [-17.3; 0.02], *P* = 0.05) and also negatively correlated with the systemic score (self-report: *R* = -0.24, *P* = 0.06; proxy-report: *R* = -0.29, *P* = 0.03) and with the height Z-score (proxy-report: *R* = -0.29, *P* = 0.03). There was no significant difference in the physical health summary scores between the different genetic subgroups. In the subgroup of 27 patients who performed a cardiopulmonary exercise test, self- and proxy-reported physical health summary scores were highly correlated with their aerobic physical capacity assessed by peak oxygen consumption (VO_2_max) and ventilatory anaerobic threshold (VAT). In the multivariate analysis, the most important independent predictors of decreased physical health were increased height, decreased body mass index, decreased VAT and use of prophylactic therapy.

**Conclusions:**

This study reports an impaired HRQoL in children and adolescents with MFS or related conditions, in comparison with matched healthy children. Educational and rehabilitation programs must be developed and evaluated to improve exercise capacity and HRQoL in these patients.

**Trial registration:**

ClinicalTrials.gov, NCT03236571. Registered 28 July 2017.

**Supplementary Information:**

The online version contains supplementary material available at 10.1186/s13023-024-03191-0.

## Background

Marfan syndrome (MFS) is an autosomal dominant genetic disorder of the connective tissue with an estimated incidence of 1 in 5,000 live births [1]. This syndrome affects multiple organs and tissues including cardiovascular (i.e., mitral valve prolapse, aortic root aneurysm and dissection), ocular (i.e., severe myopia and ectopia lentis), and skeletal (i.e., disproportionately tall stature, arachnodactyly, pectus deformity, and scoliosis) systems. MFS is caused by a pathogenic variant in the *FBN1* gene encoding fibrillin-1, inherited from one parent in nearly 75% of cases [2]. MFS diagnostic criteria are based on the 2010 revised Ghent nosology [3] that combines clinical signs, family history and molecular diagnosis. According to this nosology, the combination of an aortic root aneurysm/dissection and ectopia lentis, i.e., the two cardinal features of MFS, is sufficient to make the diagnosis. When an aortic disease is present but ectopia lentis is not, a ‘systemic score’ including all other cardiovascular and ocular manifestations of MFS and findings in other organ systems, such as the skeleton, dura, skin and lungs, guides the diagnosis. Although not mandatory, genetic testing for *FBN1* variants can also contribute to identifying pediatric forms of MFS, where age-related clinical manifestations may not be present, and also in adult forms overlapping with other heritable thoracic aortic diseases. Indeed, MFS shares cardiovascular and skeletal features with related conditions, notably Loeys-Dietz syndromes (LDS) that are linked to pathogenic variants of genes encoding components of the TGFβ signaling pathway, notably transforming growth factor beta-receptors 1 and 2 (TGFβR1 and TGFβR2) and mothers against decapentaplegic homolog 3 (SMAD3) [4].

Medical treatment (i.e., β-adrenergic receptor blocker or angiotensin II receptor blocker) to slow aortic growth and prophylactic aortic aneurysm surgery to prevent aortic dissections have led to improved lifespan in individuals with MFS [1]. According to the 2010 American Heart Association (AHA) / American College of Cardiology (ACC) Thoracic Aortic Disease guidelines, medical treatment is indicated in children with MFS from 5 years of age, as studies demonstrate that medication slows aortic dilatation, and the earlier medication is started, the greater the effect [5]. With the advances in the diagnosis and management of life-threatening cardiovascular events over the past 50 years, individuals with MFS currently have a life expectancy that approaches that of the general population [6]. However, patients with MFS continue to face many morbidities, including the sequelae of multiple surgeries as well as musculoskeletal impairments that may affect their overall health-related quality of life (HRQoL). In the past decade, most HRQoL-controlled studies in the MFS population have enrolled adult individuals and showed a significant impairment in their quality of life [7–18]. In these adult studies, psychological and social factors were more strongly correlated with HRQoL than factors related to the severity of the disease. Conversely, the few studies that have evaluated HRQoL in children and adolescents with MFS [19–22] show divergent results, with normal or impaired HRQoL in children with MFS, and were difficult to compare, notably because of different scales used to measure HRQoL. In these pediatric studies, as in most adult studies, the lack of systematic molecular diagnosis of MFS makes any genotype–phenotype correlation analysis impossible. In this cross-sectional controlled study, we aimed to assess the HRQoL of 64 children and adolescents with a molecular diagnosis of MFS or LDS in comparison with peers recruited in schools. We also sought to evaluate the factors associated with HRQoL in this population.

## Methods

### Study design

This cross-sectional controlled study was carried out between September 2018 and June 2022 in a pediatric tertiary care national reference center for MFS and related conditions (Toulouse university hospital, Occitanie region, France), and in 14 school classes in the Occitanie region, France, randomly selected from the Department of Education’s database.

### Patient population

Children and adolescents aged 5–18 years, with a molecularly confirmed diagnosis of MFS or related condition (i.e., LDS), were prospectively recruited during a medical outpatient visit at the reference center of Toulouse University Hospital. We did not include children unable to understand the HRQoL questionnaire (e.g., non-French speakers, severe neurodevelopmental disorder) or children with any recent surgical procedures (6 months prior to inclusion visit). Children filled in the HRQoL self-questionnaire under the supervision of a trained clinical research assistant, and their parents filled in the HRQoL proxy questionnaire in a separate room.

### Control population

In the 14 selected school classes, parents or legal guardians of all children aged 5 to 18 years were offered the chance to participate in the study, using the same recruitment procedure for each class, which was similar to the one used at the hospital, as previously detailed [23, 24]. Children completed the HRQoL self-questionnaire under the supervision of a clinical research assistant, at school. Parents filled in the HRQoL proxy questionnaire separately at home. Each child with MFS included in this study was matched on age and sex with 2 healthy children of this control population.

### Health-related QoL assessment

In both groups, HRQoL was assessed using the Pediatric Quality of Life Inventory (PedsQL™) generic questionnaire. The PedsQL™ is a generic pediatric HRQoL instrument widely used in healthy and chronically ill children [25]. Different age-adjusted PedsQL™ versions are available, with self-reported questionnaires to be completed by children aged above 5 years and proxy-reported questionnaires to be completed by their parents. Four versions of the PedsQL™ self and proxy questionnaires were used in this study (5–7, 8–12, 13–17 and 18–25 years old).

The 23 items in the PedsQL™ include four multidimensional scales: physical (8 items), emotional (5 items), social (5 items) and school (5 items) functioning. Each item uses a 5-point Likert scale from 0 (never) to 4 (almost always). Items are reverse scored and linearly transformed to a 0-to-100 scale, with higher scores indicating a better HRQoL. Two summary scores can be calculated: the psychosocial health summary score and the physical health summary score.

The reliability and validity of the PedsQL™ questionnaires have been demonstrated in healthy and chronically ill patient populations [25, 26]. The translation and cultural adaptation into French was performed by the MAnagement de Projets Insu (MAPI) Research Institute (www.mapi-trust.org; French project management support network), in accordance with international guidelines [27]. Our group has performed psychometric validation of the self and proxy French versions of the PedsQL™ (age range 8–12 years) [28] and shown the sensitivity of this instrument in various controlled prospective HRQoL studies among healthy controls and children with various chronic diseases [23, 24].

### Clinical outcomes in the MFS population

Personal history, as well as clinical data (including ocular, cardiovascular and skeletal features of MFS), were obtained by retrospective chart review from the French national rare disease patient database (BaMaRa). Auxological parameters (height and weight with the calculation of the bone mass index, BMI) were collected and converted into age- and sex-specific Z-scores using published reference data [29]. The ‘systemic score’ was calculated in accordance with the 2010 revised Ghent nosology. Apart from aortic disease and ectopia lentis, this score included all other MFS cardiovascular (i.e., mitral valve prolapse) and ocular (i.e., severe myopia) manifestations, as well as findings in other organ systems, such as the skeleton, dura, skin and lungs (i.e., disproportionately tall stature, arachnodactyly, pectus deformity, scoliosis, pes planus, protrusion acetabuli, pneumothorax, dural ectasia, skin striae, and facial features). A systemic score ≥ 7 was considered a major criterion for the diagnosis of MFS. Echocardiographic measurements (outflow tract diameter, aortic sinuses, sinotubular junction, and tubular ascending aorta) were also collected and converted to Z-scores [30]. Aortic dilatation was defined as aortic root Z-score > 2.

Aerobic physical capacity, a predictor of HRQoL in children with cardiovascular disease [31] was assessed in MFS patients undergoing cardiopulmonary exercise testing in their routine follow-up. A single pediatric cycle ergometer protocol was used to obtain a homogeneous incremental overall duration between 8 and 12 min, as in our previous similar studies [32, 33]. The exercise test was considered maximal when the following criteria were reached: respiratory exchange ratio (RER = VCO_2_/VO_2_) ≥ 1.05, the limit of the child’s tolerance despite verbal encouragement, and inability to provide a minimum pedaling frequency of 60 per minute despite verbal encouragement. The maximum heart rate > 85% of the maximum age-predicted heart rate was not used as a criterion for maximal effort in this study, as most MFS patients are on beta-blockers. The values of peak oxygen consumption (VO_2 max_) and ventilatory anaerobic threshold (VAT) were collected. VO_2max_ and VAT were expressed in raw values (mL/Kg/min) and normalized as a percentage of predicted VO_2max_ according to the height-based pediatric equations from Cooper et al. [34]. A VO_2 max_ < 80% of predicted values indicated impaired aerobic physical capacity [32, 33]. A lower VAT < 55% of predicted VO_2max_ values supported a finding of physical deconditioning [32, 33].

Parents of children with MFS were also questioned on their socioeconomic characteristics including education level, whether or not they were working, civil status (i.e., parents living together or divorced, death of one parent), residential area (i.e., urban or rural area, single-family home or apartment), number of children and sibling rank.

### Molecular analyses in the MFS population

Molecular analyses were performed in a single reference laboratory using next-generation sequencing (NGS) panel of 35 genes involved in MFS and related disorders, following previously reported standard procedures [2]. Where possible, familial segregation of variants was investigated. Only likely pathogenic or pathogenic variants (classes 4 and 5 according to American College of Medical Genetics and Genomics–Association for Molecular Pathology [ACMG-AMP] recommendations, [35]) were considered for this study. *FBN1* variants were categorized either as predicted to result in premature stop codon (PTC) or in-frame (i.e., haploinsufficiency/dominant negative). In-frame were also categorized as variants affecting cysteine content (missense variants that substitute for a cysteine [+Cys] or that substitute a cysteine for another amino acid [−Cys]) or not affecting cysteine content [36].

### Statistical analysis

Auxological parameters (height and BMI) and aortic sinus diameters were converted into age- and sex-specific Z-scores from the mean result in the reference population, using the published reference data cited in the description of each measurement technique. Descriptive data were presented as frequencies for categorical variables and as mean and standard deviations (SD) for continuous variables. The continuous variables distributions were tested using the Shapiro-Wilk test. Characteristics according to the pathogenic variant group (*FBN1 vs TGFβ* genes) and prophylactic cardiac therapy were compared using parametric Student’s t-test if the distribution was Gaussian or the Mann–Whitney test otherwise and with Chi-square or Fisher test.

HRQoL scores were analysed using a linear mixed model with adjustments for age and gender. Brotherhood was introduced as a random effect.

The correlations between HRQoL scores and the different continuous parameters (age, BMI, systemic score, aortic sinus diameter, and aerobic physical capacity) were evaluated using Pearson’s coefficient if their distribution were Gaussian otherwise by Spearman’s coefficient.

To identify the factors associated with HRQoL in the MFS group, a multiple linear regression analysis was performed on the two health summary self-reported PedsQL™ scores (physical and psychosocial). The best method to select variables was determined using leave-one-out cross-validation to minimized the sum of squared errors. Finally, it was a backward selection based on the corrected Akaike Information Criterion which was applied. No collinearity between variables was detected with variance inflation factors. The normality of residues in the final model was tested using the Shapiro-Wilk test.

Statistical significance was set at 0.05 and analyses were performed using the software SAS Enterprise Guide, version 7.13 (SAS Institute, Cary, NC, USA).

## Results

A total of 187 children were recruited (mean age 11.9 ± 3.8 years, 37% female), including 63 subjects in the MFS group (mean age 12.4 ± 4.1 years, 37% female), and 124 subjects in the control group (mean age 11.6 ± 3.6 years, 37% female). Both groups were similar in terms of age and gender. For the cohort of patients with MFS, all children and adolescents followed during the study period at the reference centre for Marfan syndrome of Toulouse University hospital agreed to complete the QoL questionnaires. In this MFS cohort, 27 (40%) children underwent a cardiopulmonary exercise test as part of an exercise rehabilitation programme. The main reasons for refusal to take part in this programme were distance from home to hospital and limited availability for additional follow-up visits. However, between the overall MFS cohort and the subgroup who underwent a cardiopulmonary exercise test demographic, skeletal, cardiac and ocular characteristics were similar.

### Characteristics of the study population

The main characteristics of the MFS cohort are presented in Table 1. Twenty-six patients (41%) had ectopia lentis and lens surgery was required in 14 of them (54%). Most patients (*N* = 58, 92%) received pharmacological prophylactic treatment for aortic root enlargement with beta-blockers (*N* = 53, 91%), angiotensin II receptor blockers (*N* = 2, 3%), or both treatments combined (*N* = 3, 5%). Aortic root dilatation was observed in nearly two-thirds of children (*N* = 37, 59%), with a mean aortic root Z-score of 2.3 ± 1.3. Aortic dissection was not reported in this cohort, but two patients underwent prophylactic aortic surgery. A systemic score ≥ 7 was found for nearly half of the patients (*N* = 33, 52%). Five patients had a history of pneumothorax at a median age of 13 years at the first occurrence.

**Table 1:** Population characteristics of the MFS group

	**Total cohort** (*n* = 63)	***FBN1* variant** (*n* = 55)	***TGFβ-genes related* variant** (*n* = 8)	***P*-value**^**a**^
**Female**	23 (37)	21 (38)	2 (25)	0.70
**Age** (years)	12.4 ± 4.1	12.5 ± 4.2	11.5 ± 3.6	0.52
**Ocular features**				
Myopia^b^	14/38 (37)	14/31 (45)	0/7 (0)	**0.03**
Ectopia lentis	26 (41)	26 (47)	0 (0)	**0.02**
**Cardiac features**				
Abnormal mitral valve	27 (43)	20 (36)	7 (88)	**0.02**
Aortic sinus diameter (Z-score)	2.3 ± 1.3	2.4 ± 1.3	1.8 ± 1.7	0.28
Aortic root dilatation (Z-score ≥ 2)^c^	37/61 (61)	33/54 (61)	4/7 (57)	1.00
**Skeletal features**				
Height (Z-score)	2.7 ± 1.5	2.9 ± 1.4	1.7 ± 1.5	**0.03**
Body mass index (Z-score)	-1.0 ± 1.7	-1.1 ± 1.7	-0.7 ± 1.4	0.44
Systemic score ≥ 7	33 (52)	31 (56)	2 (25)	0.14
**Cardiopulmonary exercise test parameters**	(*n* = 27)	(*n* = 24)	(*n* = 3)	
Maximum heart rate (beats/min)	162.1 ± 22.0	161.9 ± 22.2	164.3 ± 25.3	0.86
Per cent-predicted maximum heart rate (%)	78.2 ± 10.8	78.3 ± 11.0	77.7 ± 11.6	0.93
Respiratory exchange ratio	1.2 ± 0.1	1.2 ± 0.1	1.2 ± 0.0	0.45
VO_2max_ (mL/Kg/min)	32.8 ± 8.8	32.1 ± 8.9	37.9 ± 8.3	0.30
Percent-predicted VO_2max_	63.4 ± 17.4	62.5 ± 17.8	70.4 ± 14.3	0.47
Impaired VO_2max_ (< 80% of per cent-predicted VO_2max_)	23 (85)	21 (88)	2 (67)	0.39
VAT (mL/Kg/min)	18.0 ± 4.3	17.4 ± 4.0	22.4 ± 4.4	0.06
Per cent-predicted VAT	34.9 ± 8.6	34.0 ± 8.3	41.9 ± 9.0	0.14
Impaired VAT (< 55% of per cent-predicted VO_2max_)	26 (96)	23 (92)	3 (100)	1.00

Values were expressed as number of patients (%) or mean ± SD

*VAT* Ventilatory anaerobic threshold, *VO*_*2max*_ Peak oxygen consumption

^a^*P*-value comparing patients with pathogenic variants in *FBN1 versus TGFβ* genes

^b^Information available in only 38 patients

^c^Evaluated in 61 patients because 2 patients had prophylactic aortic surgery

In terms of genetic molecular diagnosis, a (likely) pathogenic variant in *FBN1* was found in most patients (*N* = 55, 87%), predicting respectively 44% of premature termination codon (PTC) and 56% of in-frame effects. Among in-frame variants of *FBN1*, 28% of these were cysteine loss variants, 6% were cysteine addition variants, 12% were non-cysteine variants and 10% were in-frame del/dup. The variant was inherited from a heterozygous parent in 60% of patients. The remaining pathogenic variants (*N* = 8, 13%) affected genes involved in the TGFβ signaling pathway (one patient with a pathogenic variant in *TGFβR1*, one in *TGFβR2*, and 5 in *SMAD3*). As previously described, compared to patients with pathogenic variants in the TGFβ signaling pathway, patients with (likely) pathogenic variants in *FBN1* were taller, had more frequent ocular impairment and less frequent mitral valve abnormality (Table 1).

A total of 27 children with MFS performed a cardiopulmonary exercise test during the study period. Overall, their aerobic physical capacity was impaired, with a mean peak oxygen consumption (VO_2_ max) of 32.8 ± 8.8 mL/Kg/min, representing 63.4 ± 17.4% of predicted values, and a mean ventilatory anaerobic threshold (VAT) of 18.0 ± 4.3 mL/Kg/min, representing 34.9 ± 8.6% of predicted values. Impaired VO_2max_ or VAT was observed in 85% and 96% of children with MFS, respectively (Table 1).

Regarding socioeconomic characteristics of the parents of the children with MFS included in this study, about half of the parents (51% of mothers and 42% of fathers) had a high school diploma, and most of them (87% of mothers and 82% of fathers) were working. One-third (35%) of families received social benefits. Seventy-one per cent of parents lived together, 22% were divorced and one parent had died due to MFS. In terms of residential location, 45% of families lived in urban areas and 55% in rural areas, 80% lived in single-family homes and 20% in apartments. The mean number of children per family was 2.5 ± 1.4 (ranging from 1 to 7) and sibling rank was on average 1.9 ± 1.1.

### HRQoL in patients with MFS compared to matched healthy controls

**Fig. 1:**
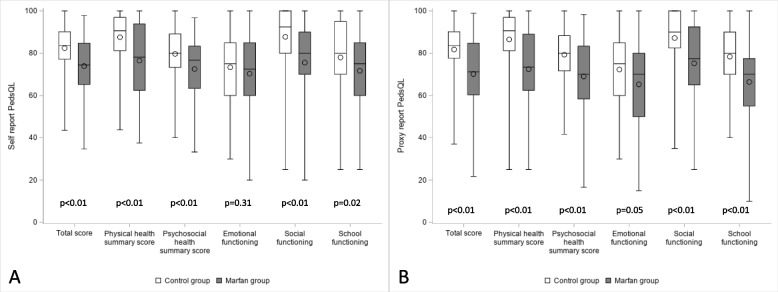
Comparison of PedsQL™ scores between patients with MFS *versus* age- and -sex-matched healthy controls. **A** Self-report, **B** Proxy-report. The bottom and top of the box represent the first and third quartiles, the band inside the box represents the second quartile (median), the circle represents the mean and the end of the whiskers represents the minimum and maximum values

HRQoL assessed by the PedsQL™ questionnaire was significantly lower in children with MFS than in age- and sex-matched healthy controls, for self-reported total scores (73.9 ± 15.1 *vs* 82.5 ± 11.9, *P* = 0.0001, absolute difference -8.7 [-12.0; -4.7], respectively), as well as for proxy-reported total scores (70.2 ± 16.4 *vs* 81.8 ± 12.4, *P* < 0.0001, absolute difference -10.9 [-15.2; -6.5], respectively) (Fig. 1, Supplemental Table 1). Similar significant differences were observed in all domains of HRQoL, except for emotional functioning, with the magnitude of the difference ranging from -10 to -5 points for self-reports, in the following dimensions (by descending order): social functioning, physical health summary score, psychosocial health summary score, and school functioning (Fig. 1, Supplemental Table 1). In proxy reports, the magnitude of the difference ranged from -16 to -10 points, by descending order, for physical score, psychosocial score, social functioning, and school functioning (Fig. 1, Supplemental Table 1).

### HRQoL predictors in patients with MFS

In the MFS group, significant associations with various parameters were observed in the PedsQL™ physical health dimension. The physical health score was significantly lower in female than in male patients (self-report: 69.5 ± 7.5 *vs* 78.3 ± 2.7, *P* = 0.04; proxy-report: 65.7 ± 3.8 *vs* 74.4 ± 3.1, *P* = 0.05). The physical health score was also negatively correlated with the systemic score (self-report: *r* = -0.24, *P* = 0.06; proxy-report: *r* = -0.29, *P* = 0.03) and with the height Z-score (proxy-report: *r* = -0.29, *P* = 0.03).

In terms of genetic molecular diagnosis, self-reported PedsQL™ social functioning was significantly lower in patients with a (likely) pathogenic *FBN1* variant than in those with a variant in genes involved in the TGFß signaling pathway (71.7 ± 3.0 *vs* 89.7 ± 7.6, *P* = 0.03, respectively). There was no significant difference in the other PedsQL™ scores according to the other *FBN1* subgroups (PTC *vs* in-frame variants, cysteine loss variant *vs* other in-frame variants, cysteine loss variant *vs* PTC).

**Fig. 2:**
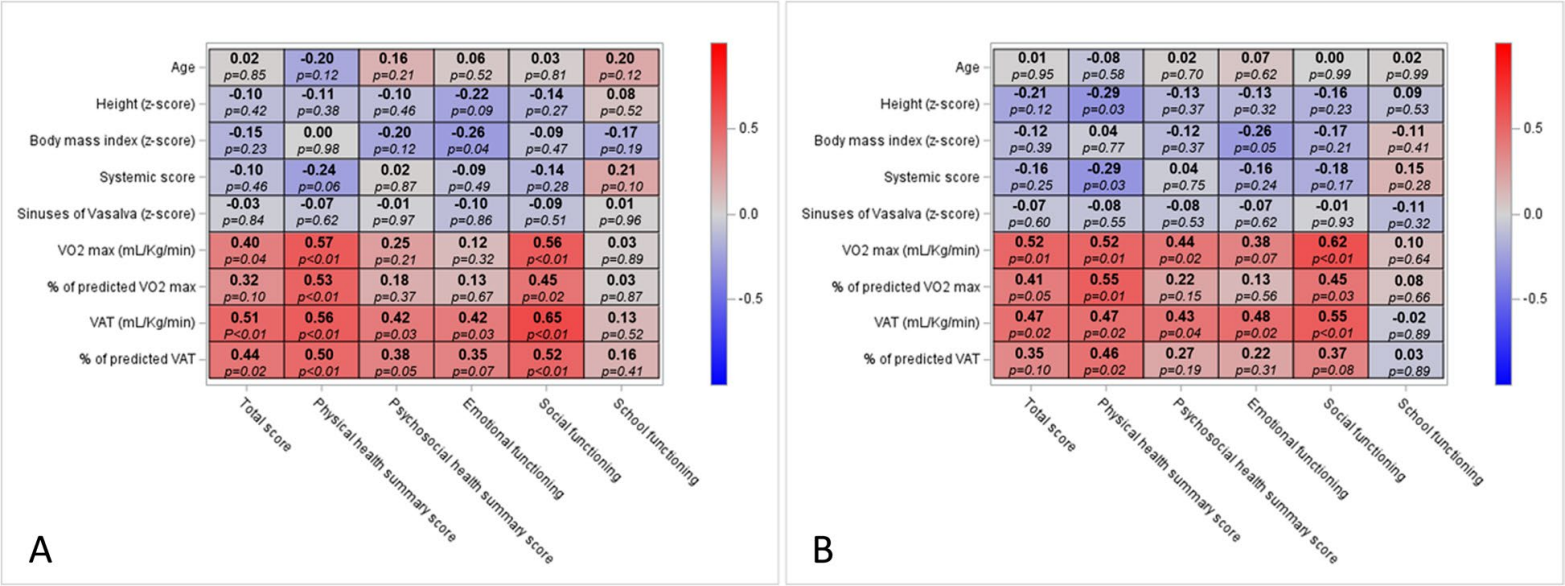
Correlation between PedsQL™ scores and clinical parameters. **A** Self-report, **B** Proxy-report

In the subgroup of 27 patients who performed a cardiopulmonary exercise test, HRQoL was associated with the level of aerobic physical capacity in children with MSF. Indeed, self- and proxy-reported PedsQL™ physical health summary and social functioning scores were highly correlated with VO_2max_ (expressed in mL/Kg/min or in the percentage of predicted VO_2max_) and VAT (expressed in mL/Kg/min or in the percentage of predicted VO_2max_) (Fig. 2).

In a multivariate analysis, the following parameters were independent predictors of a lower PedsQL™ self-reported physical health summary score: greater height (expressed in Z-score), lower body mass index (BMI) (expressed in Z-score), lower VAT (expressed in mL/Kg/min) and use of a prophylactic cardiac therapy (Table 2).

**Table 2:** Predictors of HRQoL self-reported physical functioning in children with Marfan syndrome

**Variables**	**N**	**Univariate analysis**		**Multivariable analysis (*n* = 21)**	
		**cβ [95%CI]**	***P*-value**	**aβ [95%CI]**	***P*-value**
Age (years)	62	-0.86 [-1.95; 0.25]	0.1258	-	**-**
Gender					
Female	22	8.51 [-17.80; 0.78]	0.0719	-	-
Male	40	-			
Height (z-score)	62	-0.57 [-3.74; 2.59]	**0.7185**	-4.86 [-8.71; -1.02]	**0.0164**
Body mass index (z-score)	62	0.37 [-2.33; 3.07]	**0.7855**	3.37 [0.86; 5.89]	**0.0117**
Aortic sinuses diameter (z-score)	60	-1.14 [-4.69; 2.41]	0.5213	-	**-**
Medical prophylactic therapy					
No	57	-20.00 [-35.96; -4.04]	**0.0149**	-29.19 [-46.77; -11.61]	**0.0001**
Yes	5	-		-	
Ectopia lentis					
Yes without surgery	37	1.87 [-10.33; 14.08]	0.2998	-	**-**
Yes with surgery	14	-7.87 [-19.02; 3.29]			
No	11	-			
Systemic score	62	-1.30 [-2.86; 0.26]	0.1008	-	**-**
VO_2max_ (mL/Kg/min)	27	1.01 [0.33; 1.69]	0.0053	-	**-**
VAT (mL/Kg/min)	27	2.20 [0.82; 3.57]	**0.0029**	2.96 [1.71; 4.21]	**0.0028**
Inheritance					
De novo	35	-3.02 [-13.12; 7.09]	0.5522	-	**-**
Familial	22	-		-	**-**
Place among siblings	56	-3.21 [-7.52; 1.09]	0.1406	-	**-**
Civil status					
Divorce / death	41	-10.96 [-21.01; -0.94]	0.0327	-	-
Together	16	-			
Living area					
Urban zone	26	-7.54 [-16.59; 1.51]	0.1006	-	-
Rural zone	33	-			

All variables were candidated in multivariate analysis. Variables associated with PedsQL self-reported physical functioning in both univariate and multivariable analyses are marked in bold

*cβ* Crude β, *aβ* Adjusted β, *CI* Confidence interval

The psychosocial health summary score was positively associated with civil status (parents living together *vs* divorce/death; adjusted β [95%CI] = 9.41 [0.77; 18.04], *P* = 0.03) but not with MFS characteristics.

## Discussion

In this study, we reported a significantly lower HRQoL in children and adolescents with MFS and related disorders compared to age- and sex-matched healthy children. Except for emotional function, all the other HRQoL domains (psychosocial and physical health, social and school functions) were affected. Greater height, lower body mass index, lower exercise capacity (VAT) and use of prophylactic cardiac therapy were independent predictors of a lower physical health summary score.

Only a few studies have evaluated HRQoL in the pediatric MFS population [19–22]. The results of these studies are sometimes divergent and difficult to compare notably because of differing scales used to measure HRQoL. Our results are in line with two other uncontrolled studies using the PedsQL questionnaire including respectively 11 and 256 children and adolescents with MFS [19, 20]. Consistent with our results, HRQoL was lower across multiple domains (physical, social, and school functions), except for emotional function [19]. This relative sparing of emotional function may be explained by the development of coping skills in children that allow normal emotional function despite their physical limitations. Using the Child Health Questionnaire (CHQ), Warninck et al. also reported impaired HRQoL in 74 children with MFS mainly due to impaired physical functioning, general health and negative mental health state [22]. In this study, children with MFS did not report experiencing low self-esteem and limitations in family activities. Moreover, compared to other heritable connective tissue disorders, children with MFS were less severely affected than children with hypermobile Ehlers-Danlos syndrome [22]. Only one study, which included 46 children with MFS and used the German KINDL-R instrument [21] has reported that HRQoL was unimpaired.

In this study, we also sought to identify predictors of quality of life. In a disease characterized by age-dependent progressive clinical manifestations, deterioration in HRQoL with age is to be expected. However, a comparison of prepubertal (< 12 years) with postpubertal children (> 12 years) did not reveal any worsening of HRQoL during childhood and adolescence, and age was not correlated with physical health summary scores in multiple regression analyses. Usually, older age in adult patients is significantly associated with impaired HRQoL [8, 13]. However, children may be less aware than adults of disease severity, and it is possible that the potentially life-threatening adulthood complications do not affect the daily life of pediatric patients. The stability of HRQoL during childhood and adolescence may also reflect better management and support of pediatric patients during their follow-up. Indeed, in recent years, psychological support and educational programs have been set up in our reference center with very positive feedback from patients. We drew inspiration from similar interventions in other chronic diseases, such as transition education programs, created to improve patient self-efficacy, disease knowledge, and ultimately quality of life [37].

Regarding the influence of sex-based differences, the physical health score was significantly lower in female compared to male patients using a linear mixed model with adjustment for age but this variable was no longer significant in multiple regression analyses suggesting confounding factors. In adults, it has been reported that females scored significantly lower than males on physical well-being and behavioral functioning [38]. However, in that adult study, no factors associated with lower HRQoL in female versus male patients could be identified. In pediatric studies, results were mixed. Whereas two studies did not identify sex-based differences in HRQoL [21, 22], a third reported that male sex was significantly associated with worse psychosocial HRQoL [19].

The feeling of being different from their peers due to their physical appearance may affect the quality of life of children with MFS. Indeed, in our study, clinical features such as tall stature and lean body were negatively associated with HRQoL. Accordingly, in Mueller et al. study, children with more distinct skeletal features had reduced emotional well-being subscales compared to unaffected children [21].

Surprisingly, no significant association was found between HRQoL and ocular impairment (ectopia lentis) whatever the severity, which is in line with other studies performed in children [19] and adults with MFS [7, 8, 13, 17, 18].

Genotype-phenotype correlations have been reported in MFS regarding the different cardiovascular, ocular and skeletal manifestations. Patients with predicted PTC variants of *FBN1* (leading to haploinsufficiency, quantitative defect) have more severe aortic dilatation and systemic manifestations than patients with predicted in-frame pathogenic variants (leading to a dominant negative effect, qualitative defect) [36, 39–46]. Regarding in-frame variants, variants that substitute a cysteine, which is involved in disulfide-bridge formation, for another amino acid (-Cys) are associated with more severe aortic and skeletal phenotypes whereas variants that substitute for a cysteine (+Cys) are associated with more ocular manifestations [36]. In contrast with previous pediatric and adult HRQoL studies, molecular diagnosis of MFS or related conditions was available for all patients included in this study, allowing investigation of potential genotype–phenotype correlations. Unfortunately, no significant HRQoL difference according to the different genetic subgroups was observed in this study. However, this negative result may be due to the small number of patients in each subgroup.

An original result of this study was the significant positive association of physical health score with cardiopulmonary fitness, as assessed by VO_2 max_ and VAT. Although maximum oxygen uptake (VO_2max_) reflects an integrative exercise response involving the pulmonary, cardiovascular and skeletal muscle systems, the ventilatory anaerobic threshold (VAT) is an index used to estimate exercise capacity. Patients with an impaired exercise capacity reached the VAT earlier, suggesting a higher degree of muscular deconditioning. Children and adolescents with MFS report increased pain and fatigue, and limited participation in activities and daily life, compared with their healthy peers [22, 43–46]. The same complaints are reported in adults with MFS, resulting in impaired HRQoL [11, 17]. Physical deconditioning in MFS is multifactorial and may involve the severity of the ocular, cardiac and skeletal manifestations of the disease, however the physical activity restriction recommendations by physicians and the over-protectiveness of parents are also factors. Moreover, children and adolescents with a sedentary lifestyle may be trapped in a vicious cycle of muscular deconditioning, even in the initial absence of severe physical impairment. As a result, the promotion of physical activity should be a major educational objective for these patients. Greater awareness among all professionals involved in the care of children with MFS and their families must be developed. Furthermore, specific rehabilitation programs are needed to improve the exercise capacity and consequently the quality of life of these patients, as in children with congenital heart disease [37].

Although our study is one of the largest series of children and adolescents with a molecular diagnosis of MFS assessed for quality of life, it has several limitations. There was a slight predominance of males over females (63 *vs* 37%), but this discrepancy was taken into account in the statistical analysis. In the MFS cohort, less than half (40%) of the children underwent a cardiopulmonary exercise test as part of an exercise rehabilitation programme, but there was no statistical difference between the overall MFS cohort and this subgroup. Unfortunately, the number of patients in each genetic abnormality subgroup was small, which may explain the lack of genotype-phenotype correlations. Finally, individual longitudinal follow-up with age on QoL scores would be very interesting and could be proposed in future studies.

## Conclusions

This study reports impaired HRQoL in children and adolescents with MFS, in comparison with healthy matched peers. These findings emphasize the need to monitor the HRQoL of children and adolescents with MFS. Moreover, this study established a correlation between physical fitness and quality of life, highlighting the need to develop and evaluate educational and rehabilitation programs to improve exercise capacity and HRQoL in these patients.

### Supplementary Information

**Supplementary Material 1.**

## Data Availability

The authors confirm that the aggregated data supporting the findings of this study are available within the article and its supplementary materials. Restrictions apply to the availability of raw data, which were used under limited authorization from patients and competent authorities for the present study. Data may be made available by the authors to other researchers with permission from the Toulouse University Hospital, provided all legal authorizations are obtained.

## References

[1] Milewicz DM, Braverman AC, De Backer J, Morris SA, Boileau C, Maumenee IH (2021). Marfan syndrome. Nat Rev Dis Primers.

[2] Chesneau B, Plancke A, Rolland G, Chassaing N, Coubes C, Brischoux-Boucher E (2021). Parental mosaicism in Marfan and Ehlers-Danlos syndromes and related disorders. Eur J Hum Genet.

[3] Loeys BL, Dietz HC, Braverman AC, Callewaert BL, De Backer J, Devereux RB (2010). The revised Ghent nosology for the Marfan syndrome. J Med Genet.

[4] Velchev JD, Van Laer L, Luyckx I, Dietz H, Loeys B (2021). Loeys-Dietz syndrome. Adv Exp Med Biol.

[5] Hiratzka LF, Bakris GL, Beckman JA, Bersin RM, Carr VF, Casey DE (2010). 2010 ACCF/AHA/AATS/ACR/ASA/SCA/SCAI/SIR/STS/SVM guidelines for the diagnosis and management of patients with Thoracic Aortic Disease: a report of the American College of Cardiology Foundation/American Heart Association Task Force on Practice Guidelines, American Association for Thoracic Surgery, American College of Radiology, American Stroke Association, Society of Cardiovascular Anesthesiologists, Society for Cardiovascular Angiography and Interventions, Society of Interventional Radiology, Society of Thoracic Surgeons, and Society for Vascular Medicine. Circulation.

[6] Pyeritz RE. Marfan syndrome: improved clinical history results in expanded natural history. Genet Med. 2019;21(8):1683-90. 10.1038/s41436-018-0399-4 30573797

[7] Foran JR, Pyeritz RE, Dietz HC, Sponseller PD (2005). Characterization of the symptoms associated with dural ectasia in the Marfan patient. Am J Med Genet A.

[8] Fusar-Poli P, Klersy C, Stramesi F, Callegari A, Arbustini E, Politi P (2008). Determinants of quality of life in Marfan syndrome. Psychosomatics.

[9] Ghanta RK, Green SY, Price MD, Arredondo CC, Wainwright D, Preventza O (2016). Midterm survival and quality of life after extent II thoracoabdominal aortic repair in Marfan syndrome. Ann Thorac Surg.

[10] Goldfinger JZ, Preiss LR, Devereux RB, Roman MJ, Hendershot TP, Kroner BL (2017). Marfan syndrome and quality of life in the GenTAC registry. J Am Coll Cardiol.

[11] Moon JR, Cho YA, Huh J, Kang IS, Kim DK (2016). Structural equation modeling of the quality of life for patients with marfan syndrome. Health Qual Life Outcomes.

[12] Peters KF, Horne R, Kong F, Francomano CA, Biesecker BB (2001). Living with Marfan syndrome II. Medication adherence and physical activity modification. Clin Genet.

[13] Rand-Hendriksen S, Johansen H, Semb SO, Geiran O, Stanghelle JK, Finset A (2010). Health-related quality of life in Marfan syndrome: a cross-sectional study of Short Form 36 in 84 adults with a verified diagnosis. Genet Med.

[14] Rao SS, Venuti KD, Dietz HC, Sponseller PD (2016). Quantifying health status and function in Marfan syndrome. J Surg Orthop Adv.

[15] Ratiu I, Virden TB, Baylow H, Flint M, Esfandiarei M (2018). Executive function and quality of life in individuals with Marfan syndrome. Qual Life Res.

[16] Schoormans D, Radonic T, de Witte P, Groenink M, Azim D, Lutter R (2012). Mental quality of life is related to a cytokine genetic pathway. PLoS One.

[17] Velvin G, Bathen T, Rand-Hendriksen S, Geirdal AO (2016). Satisfaction with life in adults with Marfan syndrome (MFS): associations with health-related consequences of MFS, pain, fatigue, and demographic factors. Qual Life Res.

[18] Verbraecken J, Declerck A, Van de Heyning P, De Backer W, Wouters EF (2001). Evaluation for sleep apnea in patients with Ehlers-Danlos syndrome and Marfan: a questionnaire study. Clin Genet.

[19] Handisides JC, Hollenbeck-Pringle D, Uzark K, Trachtenberg FL, Pemberton VL, Atz TW (2019). Health-related quality of life in children and young adults with Marfan syndrome. J Pediatr.

[20] Johansen H, Dammann B, Andresen IL, Fagerland MW (2013). Health-related quality of life for children with rare diagnoses, their parents’ satisfaction with life and the association between the two. Health Qual Life Outcomes.

[21] Mueller GC, Steiner K, Wild JM, Stark V, Kozlik-Feldmann R, Mir TS (2016). Health-related quality of life is unimpaired in children and adolescents with Marfan syndrome despite its distinctive phenotype. Acta Paediatr.

[22] Warnink-Kavelaars J, de Koning LE, Rombaut L, Menke LA, Alsem MW, van Oers HA (2022). Heritable connective tissue disorders in childhood: decreased health-related quality of life and mental health. Am J Med Genet A.

[23] Abassi H, Huguet H, Picot MC, Vincenti M, Guillaumont S, Auer A (2020). Health-related quality of life in children with congenital heart disease aged 5 to 7 years: a multicentre controlled cross-sectional study. Health Qual Life Outcomes.

[24] Amedro P, Dorka R, Moniotte S, Guillaumont S, Fraisse A, Kreitmann B (2015). Quality of life of children with congenital heart diseases: a multicenter controlled cross-sectional study. Pediatr Cardiol.

[25] Varni JW, Seid M, Kurtin PS (2001). PedsQL 4.0: reliability and validity of the Pediatric Quality of Life Inventory version 4.0 generic core scales in healthy and patient populations. Med Care.

[26] Varni JW, Limbers CA (2009). The pediatric quality of life inventory: measuring pediatric health-related quality of life from the perspective of children and their parents. Pediatr Clin North Am.

[27] Guillemin F, Bombardier C, Beaton D (1993). Cross-cultural adaptation of health-related quality of life measures: literature review and proposed guidelines. J Clin Epidemiol.

[28] Amedro P, Huguet H, Macioce V, Dorka R, Auer A, Guillaumont S (2021). Psychometric validation of the French self and proxy versions of the PedsQL 4.0 generic health-related quality of life questionnaire for 8–12 year-old children. Health Qual Life Outcomes.

[29] Heude B, Scherdel P, Werner A, Le Guern M, Gelbert N, Walther D (2019). A big-data approach to producing descriptive anthropometric references: a feasibility and validation study of paediatric growth charts. Lancet Digit Health.

[30] Gautier M, Detaint D, Fermanian C, Aegerter P, Delorme G, Arnoult F (2010). Nomograms for aortic root diameters in children using two-dimensional echocardiography. Am J Cardiol.

[31] Amedro P, Picot MC, Moniotte S, Dorka R, Bertet H, Guillaumont S (2016). Correlation between cardio-pulmonary exercise test variables and health-related quality of life among children with congenital heart diseases. Int J Cardiol.

[32] Amedro P, Gavotto A, Guillaumont S, Bertet H, Vincenti M, De La Villeon G (2018). Cardiopulmonary fitness in children with congenital heart diseases versus healthy children. Heart.

[33] Moreau J, Socchi F, Renoux MC, Requirand A, Abassi H, Guillaumont S, et al. Cardiopulmonary fitness in children with asthma versus healthy children. Arch Dis Child. 2022. 10.1136/archdischild-2021-323733 36446481

[34] Cooper DM, Berry C, Lamarra N, Wasserman K (1985). Kinetics of oxygen uptake and heart rate at onset of exercise in children. J Appl Physiol (1985).

[35] Richards S, Aziz N, Bale S, Bick D, Das S, Gastier-Foster J (2015). Standards and guidelines for the interpretation of sequence variants: a joint consensus recommendation of the American College of Medical Genetics and Genomics and the Association for Molecular Pathology. Genet Med.

[36] Arnaud P, Milleron O, Hanna N, Ropers J, OuldOuali N, Affoune A (2021). Clinical relevance of genotype-phenotype correlations beyond vascular events in a cohort study of 1500 Marfan syndrome patients with FBN1 pathogenic variants. Genet Med.

[37] Werner O, Bredy C, Lavastre K, Guillaumont S, De La Villeon G, Vincenti M (2021). Impact of a transition education program on health-related quality of life in pediatric patients with congenital heart disease: study design for a randomised controlled trial. Health Qual Life Outcomes.

[38] Thijssen CGE, Doze DE, Gokalp AL, Timmermans J, Peters JB, Elbers-van de Ven LHC (2020). Male-female differences in quality of life and coping style in patients with Marfan syndrome and hereditary thoracic aortic diseases. J Genet Couns.

[39] Baudhuin LM, Kotzer KE, Lagerstedt SA (2015). Decreased frequency of FBN1 missense variants in Ghent criteria-positive Marfan syndrome and characterization of novel FBN1 variants. J Hum Genet.

[40] Becerra-Munoz VM, Gomez-Doblas JJ, Porras-Martin C, Such-Martinez M, Crespo-Leiro MG, Barriales-Villa R (2018). The importance of genotype-phenotype correlation in the clinical management of Marfan syndrome. Orphanet J Rare Dis.

[41] Franken R, Teixido-Tura G, Brion M, Forteza A, Rodriguez-Palomares J, Gutierrez L (2017). Relationship between fibrillin-1 genotype and severity of cardiovascular involvement in Marfan syndrome. Heart.

[42] Rommel K, Karck M, Haverich A, von Kodolitsch Y, Rybczynski M, Muller G (2005). Identification of 29 novel and nine recurrent fibrillin-1 (FBN1) mutations and genotype-phenotype correlations in 76 patients with Marfan syndrome. Hum Mutat.

[43] Warnink-Kavelaars J, Beelen A, Dekker S, Nollet F, Menke LA, Engelbert RHH (2019). Marfan syndrome in childhood: parents’ perspectives of the impact on daily functioning of children, parents and family; a qualitative study. BMC Pediatr.

[44] Warnink-Kavelaars J, Beelen A, Goedhart T, de Koning LE, Nollet F, Alsem MW (2019). Marfan syndrome in adolescence: adolescents’ perspectives on (physical) functioning, disability, contextual factors and support needs. Eur J Pediatr.

[45] Warnink-Kavelaars J, van Oers HA, Haverman L, Buizer AI, Alsem MW, Engelbert RHH (2021). Parenting a child with Marfan syndrome: distress and everyday problems. Am J Med Genet A.

[46] Lidal IB, Bathen T, Johansen H, Velvin G (2020). A scoping review presenting a wide variety of research on paediatric and adolescent patients with Marfan syndrome. Acta Paediatr.

