# Supplementary material for: Health-related quality of life in children and adolescents with Marfan syndrome or related disorders: a controlled cross-sectional study
Source: Orphanet J Rare Dis. 2024 Apr 30;19:180. doi: 10.1186/s13023-024-03191-0 (PMC11059743; doi:10.1186/s13023-024-03191-0)
Supplement: Supplementary file 1 — Supplementary Material 1. [file 13023_2024_3191_MOESM1_ESM.docx]

**Supplemental Table 1.** Self and proxy-reported PedsQL™ scores between patients with MFS *versus* age- and gender-matched healthy controls

|  | **Self-reports** | | | | **Proxy-reports** | | | |
| --- | --- | --- | --- | --- | --- | --- | --- | --- |
|  | **MFS group** | **Healthy controls** | **Absolute difference [95% CI]** | **P-value*** | **MFS group** | **Healthy controls** | **Absolute difference [95% CI]** | **P-value*** |
| **Total score** | 73.9 ± 15.1 | 82.5 ± 11.9 | -8.7 [-12.0; -4.4] | **< 0.0001** | 70.2 ± 16.4 | 81.8 ± 12.4 | -10.9 [-15.2; -6.5] | **< 0.0001** |
| **Physical health summary score** | 76.5 ± 17.8 | 87.6 ± 12.4 | -9.4 [-15.6; -6.3] | **< 0.0001** | 72.4 ± 19.34 | 86.5 ± 16.0 | -15.6 [-18.8; -9.4] | **< 0.0001** |
| **Psychosocial health summary score** | 72.5 ± 15.8 | 79.7 ± 12.8 | -6.7 [-10.0; -1.7] | **0.0002** | 79.3 ± 12.3 | 69.0 ± 16.8 | -10.0 [-15.0; -5.0] | **< 0.0001** |
| **Emotional functioning** | 70.3 ± 18.5 | 73.3 ± 16.9 | -5.0 [-10.0; 0.0] | 0.1445 | 65.3 ± 20.5 | 72.3 ± 16.3 | -5.0 [-10.0; 0.0] | **0.0122** |
| **Social functioning** | 75.6 ± 20.6 | 87.8 ± 16.0 | -10.0 [-15.0; -5.0] | **< 0.0001** | 75.3 ± 19.5 | 87.2 ± 15.2 | -10.0 [-15.0; -5.0] | **< 0.0001** |
| **School functioning** | 71.7 ± 18.6 | 78.0 ± 17.3 | -5.0 [-10.0; 0.0] | **0.0072** | 66.4 ± 19.6 | 78.4 ± 15.3 | -10.0 [-15.0; -5.0] | **< 0.0001** |

Values were expressed as mean ± SD

* P-value was calculated using a linear mixed model adjusted on age and gender.
